# Dataset for the assessment of presence and performance in an augmented reality environment for motor imitation learning: A case-study on violinists

**DOI:** 10.1016/j.dib.2023.109663

**Published:** 2023-10-11

**Authors:** Adriaan Campo, Aleksandra Michałko, Bavo Van Kerrebroeck, Mark Leman

**Affiliations:** Institute for Psychoacoustics and Electronic Music (IPEM), Ghent University, Miriam Makebaplein 1, 9000, Ghent, Belgium

**Keywords:** Music technology, Augmented reality, Presence, Motor imitation learning

## Abstract

This dataset comprises motion capture, audio, and questionnaire data from violinists who underwent four augmented reality training sessions spanning a month. The motion capture data was meticulously recorded using a 42-marker Qualisys Animation marker set, capturing movement at a high rate of 120 Hz. Audio data was captured using two condenser microphones, boasting a bit depth of 24 and a sampling rate of 48 kHz. The dataset encompasses recordings from 2 violin orchestra section leaders and 11 participants.

Initially, we collected motion capture (MoCap) and audio data from the section leaders, who performed 2 distinct musical pieces. These recordings were then utilized to create 2 avatars, each representing a section leader and their respective musical piece. Subsequently, each avatar was assigned to a group of violinists, forming groups of 5 and 6 participants. Throughout the experiment, participants rehearsed one piece four times using a 2D representation of the avatar, and the other piece four times using a 3D representation.

During the practice sessions, participants were instructed to meticulously replicate the avatar's bowing techniques, encompassing gestures related to bowing, articulation, and dynamics. For each trial, we collected motion capture, audio data, and self-reported questionnaires from all participants. The questionnaires included the Witmer presence questionnaire, a subset of the Makransky presence questionnaire, the sense of musical agency questionnaire, as well as open-ended questions for participants to express their thoughts and experiences.

Additionally, participants completed the Immersive Tendencies questionnaire, the Music Sophistication Index questionnaire, and provided demographic information before the first session commenced.

Specifications Table

**Table:** 

Subject	Developmental and Educational Psychology
Specific subject area	Presence, performance and learning assessment in a 2D and 3D augmented reality music education application
Type of data	Table Music score Audio-file
How the data were acquired	1. Motion capture data Obtained with a Qualisys motion capture system, using 18 infrared OQUS cameras and 42 reflective markers, using the Animation marker set, a violin bow (3 markers) and a violin (3–4 markers), recorded at 120 Hz. 2. Audio Obtained with a Y-pair of condenser microphones in Ableton and synchronized with Motion Capture data using a SMTPE system, recorded at 48 kHz, and bit depth of 24. 3. Questionnaires Completed before the first trial, after each trial, or after the last trial, in LimeSurvey. 3.1. Before the first trial: - Immersive tendencies questionnaire (ITQ) - Musical sophistication index (MSI) - Demographic questions (DQ) 3.2. After every trial: - Witmer Presence questionnaire (WPQ) - Makransky Multimodal Presence questionnaire (MPQ) - Sense of Musical Agency questionnaire (SOMA) - Open questions (OQ) 3.3. After the last trial - Open questions (OQ)
Data format	Raw Filtered Analyzed
Description of data collection	Eleven amateur violin players from a student orchestra took part in a study where they practiced two previously unfamiliar pieces with an avatar through an augmented reality application. The avatars used in the application were generated from data provided by two section leaders who also served as the
	section leaders for the first and second violin sections. The avatars were presented in two different formats: 2D and 3D renderings. During the span of one month, the amateur players engaged in four practice sessions with the application, focusing on two pieces. Throughout the experiment, the pieces were performed either by a 2D or a 3D avatar. It is worth noting that the participants learned the pieces during the study and had almost no prior experience with them.
Data source location	- Institution: Ghent University, Institute for psychoacoustics and electronic music (IPEM) - City/Town/Region: Miriam Makebaplein 1, 9000 Ghent - Country: Belgium - Latitude and longitude: 51.04876912686138, 3.7286443830242963
Data accessibility	Repository name: Zenodo Data identification number: 10.5281/zenodo.8147435 Direct URL to data: https://doi.org/10.5281/zenodo.8147435
Related research article	Adriaan Campo, Aleksandra Michałko, Bavo Van Kerrebroeck, Boris Stajic, Maja Pokric, Marc Leman, The assessment of presence and performance in an AR environment for motor imitation learning: A case-study on violinists, Computers in Human Behavior, 2023, 107810, ISSN 0747-5632, https://doi.org/10.1016/j.chb.2023.107810.

## 1 Value of the Data

• These data investigate performance, feeling of presence, and learning process of a small group of violinists, who use augmented reality with different layers of reality/stereoscopic information to master a musical piece. This dataset is unique, because it assesses kinematics and audio of musicians performing a complex task in different states of presence and immersion, while using augmented reality.
• These data are of interest for the scientific community studying behavior in augmented reality, the interplay between presence and learning, the interplay between immersion and presence, and motor imitation learning.
• The data can be used to study kinematics and audio of musicians rehearsing a piece, and to study interaction of performance, feeling of presence, and learning process in an augmented reality environment.

## 2 Objective

This study aims to address the educational challenge of acquiring sophisticated gestures. The dataset collected for this study is utilized to investigate the impact of practicing with an augmented reality (AR) play-along application on playing performance, learning process, and feeling of presence.

The study consists of two experimental conditions: one involving a 2D simulation of a virtual section leader and the other utilizing a 3D simulation within a virtual HoloLens 2 environment. Eleven participants engaged in four practice trials spaced evenly over the course of a month, with each participant experiencing both conditions in a within-subject design.

Like the dynamics of real orchestral playing, participants were instructed to closely imitate the avatar's bow movements, encompassing bowings, articulations, and dynamics. The study recorded and analyzed violin playing gestures using kinematic metrics. Additionally, questionnaires were administered to explore subjective experiences of presence and establish participants' musicality and immersive tendencies.

By employing hierarchical regression modeling, the study examines whether the play-along application's conditions influenced gesture similarity, imitation learning, and feelings of presence. This analysis provides valuable insights into the effects of different experimental conditions on skill acquisition and immersive experiences.

## 3 Data Description

### 3.1 Repository structure

The repository contains several zip files, which contain a set of individual data files. We describe the general content of every zip file and supplement this information with a table describing the individual data files in the zip file. An overview of the different zip-files is given in Table 1.

**Table 1:** Overview of different zip-files in repository.

Zip-files in repository	description
Labeled_MoCap_Data.zip	Labeled motion capture data
Joint_Angle_Data.zip	Joint angles extracted from mocap data
Analyzed_Data.zip	Filtered and analyzed mocap data
Audio_Data.zip	Audio data participants
Questionnaire_Data.zip	Questionnaire data
Scores.zip	Scores played by participants
Avatar_Data.zip	All data collected for the avatars

### 3.2 Labeled_MoCap_Data.zip

**Fig. 1:**
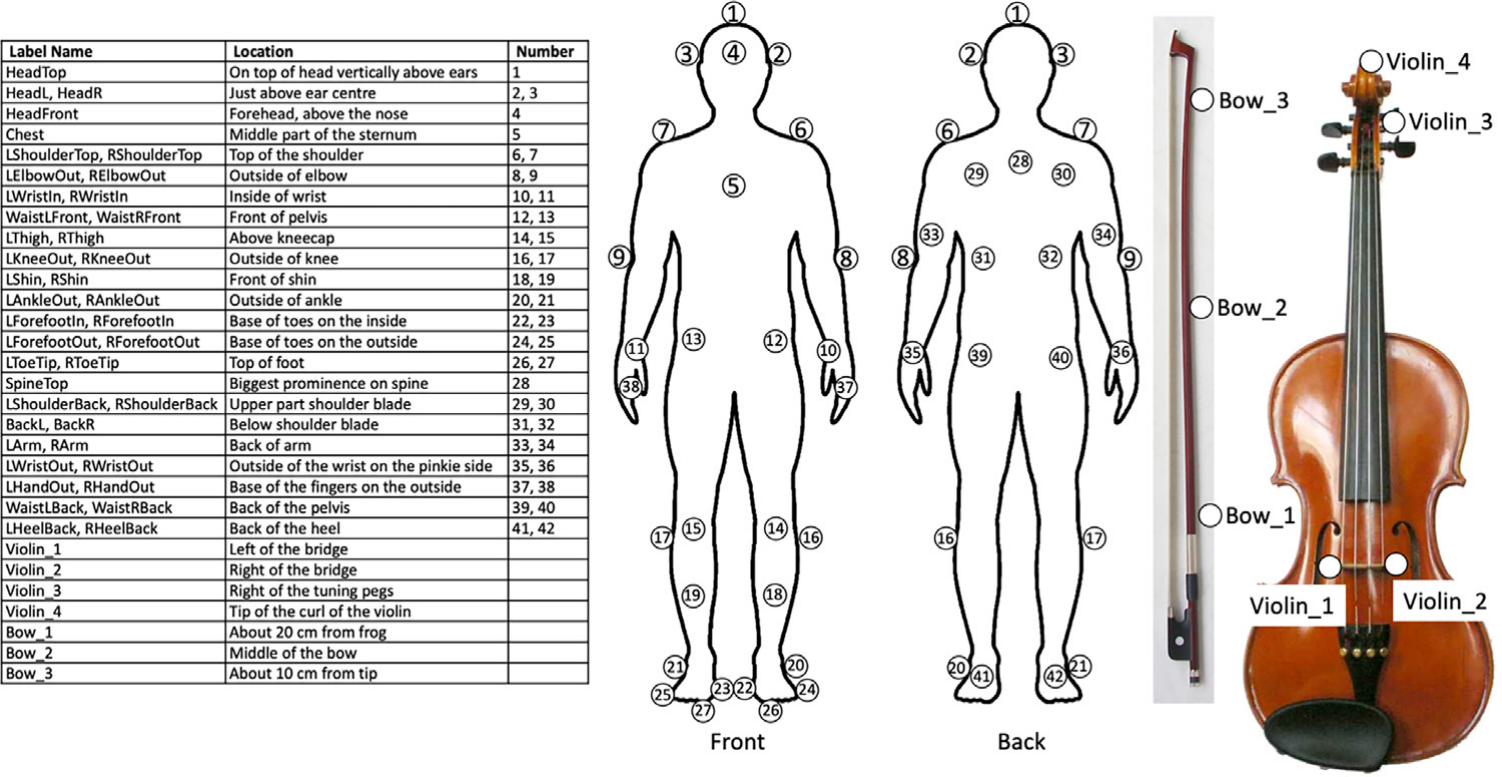
Overview of marker placement in participant, violin, and bow. Markers are indicated as white dots with a black edge.

Csv format with labeled MoCap Data, including data labels. Every column is a data stream from a marker. Marker positions are indicated in Fig. 1. Every marker has 3 data streams, referring to the x, y, and z coordinates of the marker position. In addition to the markers as indicated in Fig. 1, the violin (3–4 markers) and the violin bow (3 markers) are labelled as well. An overview of the different labels and their meaning is given in Table 2. One data file per participant (P001-P011), per trial (T1-T4), per condition (2D/3D) is presented. Additionally, the data type (MoCap), and the performed fragment (F1-F4) are given in the filename. An example of a file name is e.g., ‘P001_T1_2D_F1_MoCap.csv’ for a participant (see Table 2).

**Table 2:** Content and file structure of Labeled_MoCap_Data.zip.

File name	Labeled_MoCap_Data.zip				
Content	Participant	Trial	Condition	Piece	Data Type
P001_T1_2D_F2_MoCap.csv	1	1	2	2	Labeled MoCap Data
P001_T2_2D_F2_MoCap.csv	1	2	2	2	
P001_T3_2D_F2_MoCap.csv	1	3	2	2	
P001_T4_2D_F2_MoCap.csv	1	4	2	2	
P001_T1_3D_F1_MoCap.csv	1	1	3	1	
P001_T2_3D_F1_MoCap.csv	1	2	3	1	
P001_T3_3D_F1_MoCap.csv	1	3	3	1	
P001_T4_3D_F1_MoCap.csv	1	4	3	1	
P002_T1_2D_F1_MoCap.csv	2	1	2	1	
P002_T2_2D_F1_MoCap.csv	2	2	2	1	
P002_T3_2D_F1_MoCap.csv	2	3	2	1	
P002_T4_2D_F1_MoCap.csv	2	4	2	1	
P002_T1_3D_F2_MoCap.csv	2	1	3	2	
P002_T2_3D_F2_MoCap.csv	2	2	3	2	
P002_T3_3D_F2_MoCap.csv	2	3	3	2	
P002_T4_3D_F2_MoCap.csv	2	4	3	2	
P003_T1_2D_F3_MoCap.csv	3	1	2	3	
P003_T2_2D_F3_MoCap.csv	3	2	2	3	
P003_T3_2D_F3_MoCap.csv	3	3	2	3	
P003_T4_2D_F3_MoCap.csv	3	4	2	3	
P003_T1_3D_F4_MoCap.csv	3	1	3	4	
P003_T2_3D_F4_MoCap.csv	3	2	3	4	
P003_T3_3D_F4_MoCap.csv	3	3	3	4	
P003_T4_3D_F4_MoCap.csv	3	4	3	4	
P004_T1_2D_F1_MoCap.csv	4	1	2	1	
P004_T2_2D_F1_MoCap.csv	4	2	2	1	
P004_T3_2D_F1_MoCap.csv	4	3	2	1	
P004_T4_2D_F1_MoCap.csv	4	4	2	1	
P004_T1_3D_F2_MoCap.csv	4	1	3	2	
P004_T2_3D_F2_MoCap.csv	4	2	3	2	
P004_T3_3D_F2_MoCap.csv	4	3	3	2	
P004_T4_3D_F2_MoCap.csv	4	4	3	2	
P005_T1_2D_F3_MoCap.csv	5	1	2	3	
P005_T2_2D_F3_MoCap.csv	5	2	2	3	
P005_T3_2D_F3_MoCap.csv	5	3	2	3	
P005_T4_2D_F3_MoCap.csv	5	4	2	3	
P005_T1_3D_F4_MoCap.csv	5	1	3	4	
P005_T2_3D_F4_MoCap.csv	5	2	3	4	
P005_T3_3D_F4_MoCap.csv	5	3	3	4	
P005_T4_3D_F4_MoCap.csv	5	4	3	4	
P006_T1_2D_F1_MoCap.csv	6	1	2	1	
P006_T2_2D_F1_MoCap.csv	6	2	2	1	
P006_T3_2D_F1_MoCap.csv	6	3	2	1	
P006_T4_2D_F1_MoCap.csv	6	4	2	1	
P006_T1_3D_F2_MoCap.csv	6	1	3	2	
P006_T2_3D_F2_MoCap.csv	6	2	3	2	
P006_T3_3D_F2_MoCap.csv	6	3	3	2	
P006_T4_3D_F2_MoCap.csv	6	4	3	2	
P007_T1_2D_F4_MoCap.csv	7	1	2	4	
P007_T2_2D_F4_MoCap.csv	7	2	2	4	
P007_T3_2D_F4_MoCap.csv	7	3	2	4	
P007_T4_2D_F4_MoCap.csv	7	4	2	4	
P007_T1_3D_F3_MoCap.csv	7	1	3	3	
P007_T2_3D_F3_MoCap.csv	7	2	3	3	
P007_T3_3D_F3_MoCap.csv	7	3	3	3	
P007_T4_3D_F3_MoCap.csv	7	4	3	3	
P008_T1_2D_F4_MoCap.csv	8	1	2	4	
P008_T2_2D_F4_MoCap.csv	8	2	2	4	
P008_T3_2D_F4_MoCap.csv	8	3	2	4	
P008_T4_2D_F4_MoCap.csv	8	4	2	4	
P008_T1_3D_F3_MoCap.csv	8	1	3	3	
P008_T2_3D_F3_MoCap.csv	8	2	3	3	
P008_T3_3D_F3_MoCap.csv	8	3	3	3	
P008_T4_3D_F3_MoCap.csv	8	4	3	3	
P009_T1_2D_F1_MoCap.csv	9	1	2	1	
P009_T2_2D_F1_MoCap.csv	9	2	2	1	
P009_T3_2D_F1_MoCap.csv	9	3	2	1	
P009_T4_2D_F1_MoCap.csv	9	4	2	1	
P009_T1_3D_F2_MoCap.csv	9	1	3	2	
P009_T2_3D_F2_MoCap.csv	9	2	3	2	
P009_T3_3D_F2_MoCap.csv	9	3	3	2	
P009_T4_3D_F2_MoCap.csv	9	4	3	2	
P010_T1_2D_F2_MoCap.csv	10	1	2	2	
P010_T2_2D_F2_MoCap.csv	10	2	2	2	
P010_T3_2D_F2_MoCap.csv	10	3	2	2	
P010_T4_2D_F2_MoCap.csv	10	4	2	2	
P010_T1_3D_F1_MoCap.csv	10	1	3	1	
P010_T2_3D_F1_MoCap.csv	10	2	3	1	
P010_T3_3D_F1_MoCap.csv	10	3	3	1	
P010_T4_3D_F1_MoCap.csv	10	4	3	1	
P011_T1_2D_F4_MoCap.csv	11	1	2	4	
P011_T2_2D_F4_MoCap.csv	11	2	2	4	
P011_T3_2D_F4_MoCap.csv	11	3	2	4	
P011_T4_2D_F4_MoCap.csv	11	4	2	4	
P011_T1_3D_F3_MoCap.csv	11	1	3	3	
P011_T2_3D_F3_MoCap.csv	11	2	3	3	
P011_T3_3D_F3_MoCap.csv	11	3	3	3	
P011_T4_3D_F3_MoCap.csv	11	4	3	3	

### 3.3 Joint_Angle_Data.zip

**Fig. 2:**
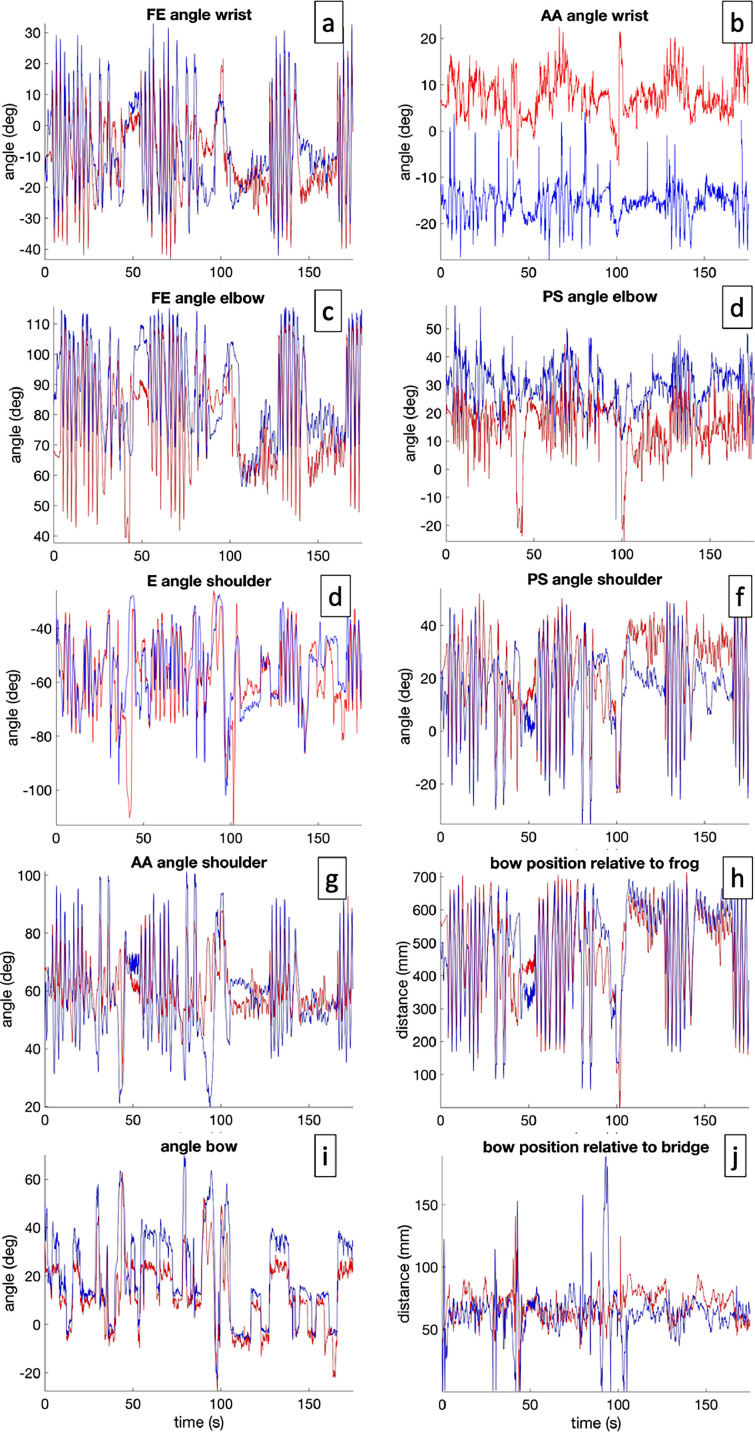
Example of joint angle data of both avatar (blue) and a participant (red) of the first violin section, playing Dvorak (fragment 1). Data represent wrist (a,b), elbow (c,d) and shoulder angles (e–g), as well as the distance between contact point between bow and string, and the frog (h) and the bridge (j), respectively, and the tilting angle between the bow and the violin (i). AA = adduction-abduction, FE = flexion-extension, PS = pronation-supination, E = elevation. (For interpretation of the references to color in this figure legend, the reader is referred to the web version of this article.)

Csv format with joint angles, including data labels. Every column is a data stream from a joint (see Fig. 2). Every joint has a varying number of data streams, depending on the calculated angles. In addition to joint angles, the angles of the instrument relative to the body are given as well, the distances of the bow to the bridge, and to distances of the bow to the strings, respectively. An overview of the different labels and their meaning is given in Table 3. One data file per participant (P001-P011), per trial (T1-T4), per condition (2D/3D) is presented. Additionally, the data type (JointAngles), and the performed fragment (F1-F4) are given in the filename. An example of a file name is e.g., ‘P001_T1_2D_F1_JointAngles.csv’ for a participant (see Table 3).

**Table 3:** Content and file structure of Joint_Angle_Data.zip.

File name	Joint_Angle_Data.zip				
Content	Participant	Trial	Condition	Piece	Data Type
P001_T1_2D_F2_JointAngles.csv	1	1	2	2	Joint Angle Data
P001_T2_2D_F2_JointAngles.csv	1	2	2	2	
P001_T3_2D_F2_JointAngles.csv	1	3	2	2	
P001_T4_2D_F2_JointAngles.csv	1	4	2	2	
P001_T1_3D_F1_JointAngles.csv	1	1	3	1	
P001_T2_3D_F1_JointAngles.csv	1	2	3	1	
P001_T3_3D_F1_JointAngles.csv	1	3	3	1	
P001_T4_3D_F1_JointAngles.csv	1	4	3	1	
P002_T1_2D_F1_JointAngles.csv	2	1	2	1	
P002_T2_2D_F1_JointAngles.csv	2	2	2	1	
P002_T3_2D_F1_JointAngles.csv	2	3	2	1	
P002_T4_2D_F1_JointAngles.csv	2	4	2	1	
P002_T1_3D_F2_JointAngles.csv	2	1	3	2	
P002_T2_3D_F2_JointAngles.csv	2	2	3	2	
P002_T3_3D_F2_JointAngles.csv	2	3	3	2	
P002_T4_3D_F2_JointAngles.csv	2	4	3	2	
P003_T1_2D_F3_JointAngles.csv	3	1	2	3	
P003_T2_2D_F3_JointAngles.csv	3	2	2	3	
P003_T3_2D_F3_JointAngles.csv	3	3	2	3	
P003_T4_2D_F3_JointAngles.csv	3	4	2	3	
P003_T1_3D_F4_JointAngles.csv	3	1	3	4	
P003_T2_3D_F4_JointAngles.csv	3	2	3	4	
P003_T3_3D_F4_JointAngles.csv	3	3	3	4	
P003_T4_3D_F4_JointAngles.csv	3	4	3	4	
P004_T1_2D_F1_JointAngles.csv	4	1	2	1	
P004_T2_2D_F1_JointAngles.csv	4	2	2	1	
P004_T3_2D_F1_JointAngles.csv	4	3	2	1	
P004_T4_2D_F1_JointAngles.csv	4	4	2	1	
P004_T1_3D_F2_JointAngles.csv	4	1	3	2	
P004_T2_3D_F2_JointAngles.csv	4	2	3	2	
P004_T3_3D_F2_JointAngles.csv	4	3	3	2	
P004_T4_3D_F2_JointAngles.csv	4	4	3	2	
P005_T1_2D_F3_JointAngles.csv	5	1	2	3	
P005_T2_2D_F3_JointAngles.csv	5	2	2	3	
P005_T3_2D_F3_JointAngles.csv	5	3	2	3	
P005_T4_2D_F3_JointAngles.csv	5	4	2	3	
P005_T1_3D_F4_JointAngles.csv	5	1	3	4	
P005_T2_3D_F4_JointAngles.csv	5	2	3	4	
P005_T3_3D_F4_JointAngles.csv	5	3	3	4	
P005_T4_3D_F4_JointAngles.csv	5	4	3	4	
P006_T1_2D_F1_JointAngles.csv	6	1	2	1	
P006_T2_2D_F1_JointAngles.csv	6	2	2	1	
P006_T3_2D_F1_JointAngles.csv	6	3	2	1	
P006_T4_2D_F1_JointAngles.csv	6	4	2	1	
P006_T1_3D_F2_JointAngles.csv	6	1	3	2	
P006_T2_3D_F2_JointAngles.csv	6	2	3	2	
P006_T3_3D_F2_JointAngles.csv	6	3	3	2	
P006_T4_3D_F2_JointAngles.csv	6	4	3	2	
P007_T1_2D_F4_JointAngles.csv	7	1	2	4	
P007_T2_2D_F4_JointAngles.csv	7	2	2	4	
P007_T3_2D_F4_JointAngles.csv	7	3	2	4	
P007_T4_2D_F4_JointAngles.csv	7	4	2	4	
P007_T1_3D_F3_JointAngles.csv	7	1	3	3	
P007_T2_3D_F3_JointAngles.csv	7	2	3	3	
P007_T3_3D_F3_JointAngles.csv	7	3	3	3	
P007_T4_3D_F3_JointAngles.csv	7	4	3	3	
P008_T1_2D_F4_JointAngles.csv	8	1	2	4	
P008_T2_2D_F4_JointAngles.csv	8	2	2	4	
P008_T3_2D_F4_JointAngles.csv	8	3	2	4	
P008_T4_2D_F4_JointAngles.csv	8	4	2	4	
P008_T1_3D_F3_JointAngles.csv	8	1	3	3	
P008_T2_3D_F3_JointAngles.csv	8	2	3	3	
P008_T3_3D_F3_JointAngles.csv	8	3	3	3	
P008_T4_3D_F3_JointAngles.csv	8	4	3	3	
P009_T1_2D_F1_JointAngles.csv	9	1	2	1	
P009_T2_2D_F1_JointAngles.csv	9	2	2	1	
P009_T3_2D_F1_JointAngles.csv	9	3	2	1	
P009_T4_2D_F1_JointAngles.csv	9	4	2	1	
P009_T1_3D_F2_JointAngles.csv	9	1	3	2	
P009_T2_3D_F2_JointAngles.csv	9	2	3	2	
P009_T3_3D_F2_JointAngles.csv	9	3	3	2	
P009_T4_3D_F2_JointAngles.csv	9	4	3	2	
P010_T1_2D_F2_JointAngles.csv	10	1	2	2	
P010_T2_2D_F2_JointAngles.csv	10	2	2	2	
P010_T3_2D_F2_JointAngles.csv	10	3	2	2	
P010_T4_2D_F2_JointAngles.csv	10	4	2	2	
P010_T1_3D_F1_JointAngles.csv	10	1	3	1	
P010_T2_3D_F1_JointAngles.csv	10	2	3	1	
P010_T3_3D_F1_JointAngles.csv	10	3	3	1	
P010_T4_3D_F1_JointAngles.csv	10	4	3	1	
P011_T1_2D_F4_JointAngles.csv	11	1	2	4	
P011_T2_2D_F4_JointAngles.csv	11	2	2	4	
P011_T3_2D_F4_JointAngles.csv	11	3	2	4	
P011_T4_2D_F4_JointAngles.csv	11	4	2	4	
P011_T1_3D_F3_JointAngles.csv	11	1	3	3	
P011_T2_3D_F3_JointAngles.csv	11	2	3	3	
P011_T3_3D_F3_JointAngles.csv	11	3	3	3	
P011_T4_3D_F3_JointAngles.csv	11	4	3	3	

### 3.4 Analyzed_Data.zip

**Fig. 3:**
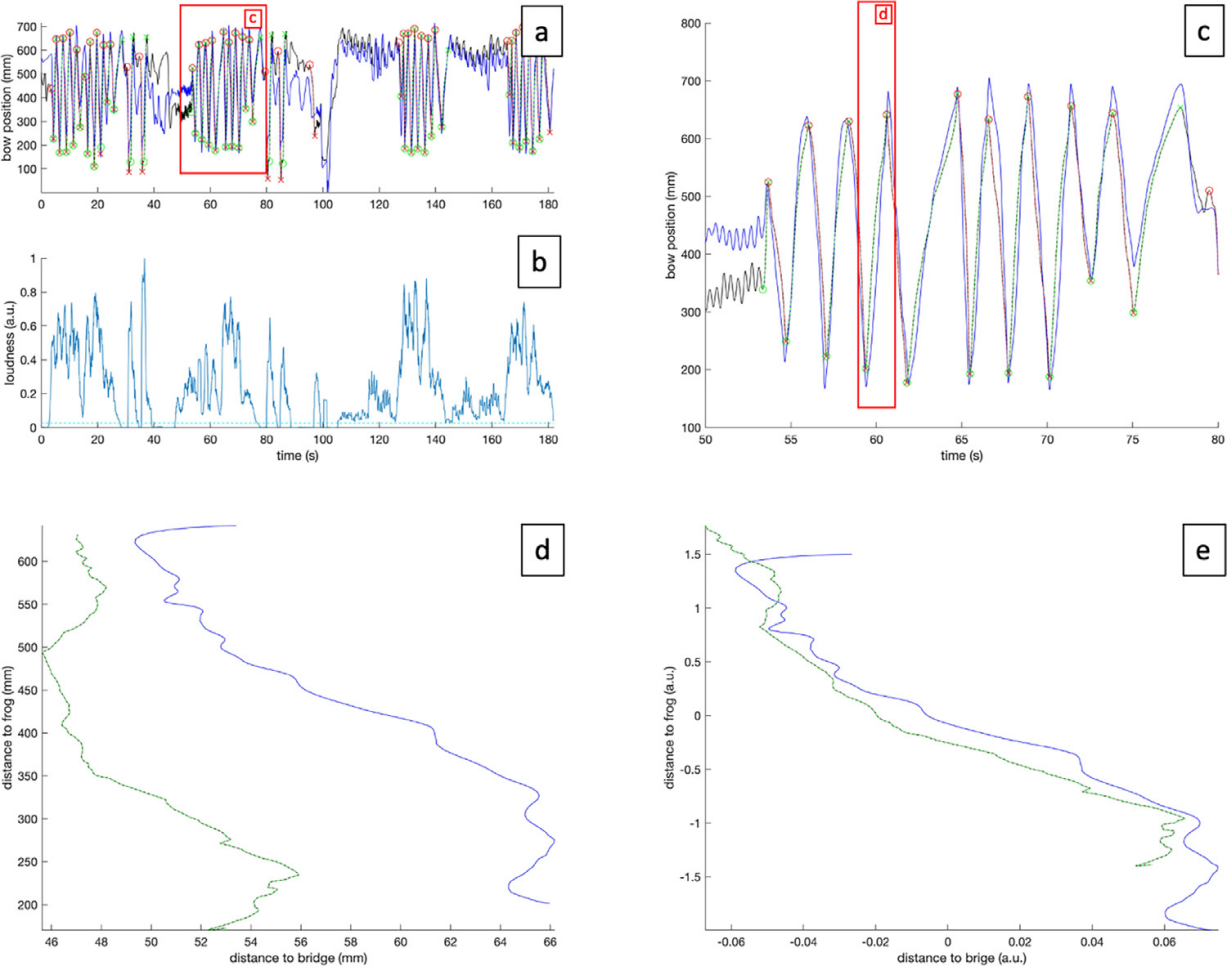
pipeline illustrating time series comparison between participant and avatar. (a) represents the bow position of avatar (black) and participant (blue). Green and red dotted lines represent analyzed downstrokes and upstrokes, respectively. (b) Represents the loudness of the performance, the green dotted line represents the loudness threshold for further analysis. (c) Displays a close-up of the red area in (a). (d) represents a 2D plot of the bow position relative to the bridge (horizontal axis) and the bow position relative to the frog (vertical axis). (e) Alignment of both signals in (d) before Procrustes distance calculation of the 2D signal. (d) Displays a close-up of the red area in (c). (For interpretation of the references to color in this figure legend, the reader is referred to the web version of this article.)

**Fig. 4:**
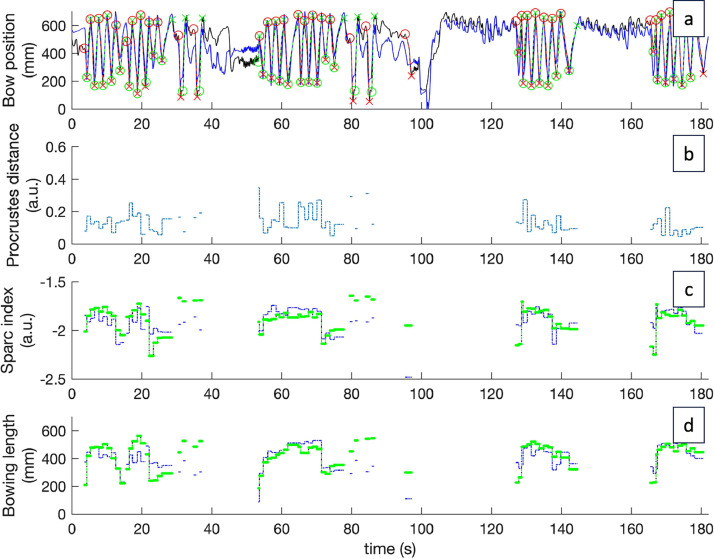
example of analyzed data. (a) Represents the bow position of avatar (black) and participant (blue). Green and red dotted lines represent analyzed downstrokes and upstrokes, respectively. (b) Displays the computed Procrustes distance (PD), of the participant bow strokes as compared to the avatar, and (c,d) displays the SPARC index and bowing length of every participant (dotted blue line) and avatar (dotted green line) bow stroke. (For interpretation of the references to color in this figure legend, the reader is referred to the web version of this article.)

Time series of filtered and analyzed data. The distances of the bow to the bridge and frog, are filtered so that only bow strokes with a certain bowing length and a certain loudness level are retained. The resulting collection of regions-of-interest (ROIs) is then analyzed for movement smoothness (as assessed with the SPARC index [1]), and a comparison is made between the profile of avatar bowing movements and participant bowing movements by means of the Procrustes distance [2] (see Fig. 3). These data are presented as csv files with 4 columns: SPARC index per ROI, Procrustes distance between the bow movement of the avatar and participant, index of start and end of each ROI (see Fig. 4). One data file per participant (P001-P011), per trial (T1-T4), per condition (2D/3D) is presented. Additionally, the data type (AnalyzedData), and the performed fragment (F1-F4) are given in the filename. An example of a file name is e.g., ‘P001_T1_2D_F1_ AnalyzedData.csv’ for a participant (see Table 4).

**Table 4:** Content and file structure of Analyzed_Data.zip.

File name	Analyzed_Data.zip				
Content	Participant	Trial	Condition	Piece	Data Type
P001_T1_2D_F2_AnalyzedData.csv	1	1	2	2	Analyzed Data
P001_T2_2D_F2_AnalyzedData.csv	1	2	2	2	
P001_T3_2D_F2_AnalyzedData.csv	1	3	2	2	
P001_T4_2D_F2_AnalyzedData.csv	1	4	2	2	
P001_T1_3D_F1_AnalyzedData.csv	1	1	3	1	
P001_T2_3D_F1_AnalyzedData.csv	1	2	3	1	
P001_T3_3D_F1_AnalyzedData.csv	1	3	3	1	
P001_T4_3D_F1_AnalyzedData.csv	1	4	3	1	
P002_T1_2D_F1_AnalyzedData.csv	2	1	2	1	
P002_T2_2D_F1_AnalyzedData.csv	2	2	2	1	
P002_T3_2D_F1_AnalyzedData.csv	2	3	2	1	
P002_T4_2D_F1_AnalyzedData.csv	2	4	2	1	
P002_T1_3D_F2_AnalyzedData.csv	2	1	3	2	
P002_T2_3D_F2_AnalyzedData.csv	2	2	3	2	
P002_T3_3D_F2_AnalyzedData.csv	2	3	3	2	
P002_T4_3D_F2_AnalyzedData.csv	2	4	3	2	
P003_T1_2D_F3_AnalyzedData.csv	3	1	2	3	
P003_T2_2D_F3_AnalyzedData.csv	3	2	2	3	
P003_T3_2D_F3_AnalyzedData.csv	3	3	2	3	
P003_T4_2D_F3_AnalyzedData.csv	3	4	2	3	
P003_T1_3D_F4_AnalyzedData.csv	3	1	3	4	
P003_T2_3D_F4_AnalyzedData.csv	3	2	3	4	
P003_T3_3D_F4_AnalyzedData.csv	3	3	3	4	
P003_T4_3D_F4_AnalyzedData.csv	3	4	3	4	
P004_T1_2D_F1_AnalyzedData.csv	4	1	2	1	
P004_T2_2D_F1_AnalyzedData.csv	4	2	2	1	
P004_T3_2D_F1_AnalyzedData.csv	4	3	2	1	
P004_T4_2D_F1_AnalyzedData.csv	4	4	2	1	
P004_T1_3D_F2_AnalyzedData.csv	4	1	3	2	
P004_T2_3D_F2_AnalyzedData.csv	4	2	3	2	
P004_T3_3D_F2_AnalyzedData.csv	4	3	3	2	
P004_T4_3D_F2_AnalyzedData.csv	4	4	3	2	
P005_T1_2D_F3_AnalyzedData.csv	5	1	2	3	
P005_T2_2D_F3_AnalyzedData.csv	5	2	2	3	
P005_T3_2D_F3_AnalyzedData.csv	5	3	2	3	
P005_T4_2D_F3_AnalyzedData.csv	5	4	2	3	
P005_T1_3D_F4_AnalyzedData.csv	5	1	3	4	
P005_T2_3D_F4_AnalyzedData.csv	5	2	3	4	
P005_T3_3D_F4_AnalyzedData.csv	5	3	3	4	
P005_T4_3D_F4_AnalyzedData.csv	5	4	3	4	
P006_T1_2D_F1_AnalyzedData.csv	6	1	2	1	
P006_T2_2D_F1_AnalyzedData.csv	6	2	2	1	
P006_T3_2D_F1_AnalyzedData.csv	6	3	2	1	
P006_T4_2D_F1_AnalyzedData.csv	6	4	2	1	
P006_T1_3D_F2_AnalyzedData.csv	6	1	3	2	
P006_T2_3D_F2_AnalyzedData.csv	6	2	3	2	
P006_T3_3D_F2_AnalyzedData.csv	6	3	3	2	
P006_T4_3D_F2_AnalyzedData.csv	6	4	3	2	
P007_T1_2D_F4_AnalyzedData.csv	7	1	2	4	
P007_T2_2D_F4_AnalyzedData.csv	7	2	2	4	
P007_T3_2D_F4_AnalyzedData.csv	7	3	2	4	
P007_T4_2D_F4_AnalyzedData.csv	7	4	2	4	
P007_T1_3D_F3_AnalyzedData.csv	7	1	3	3	
P007_T2_3D_F3_AnalyzedData.csv	7	2	3	3	
P007_T3_3D_F3_AnalyzedData.csv	7	3	3	3	
P007_T4_3D_F3_AnalyzedData.csv	7	4	3	3	
P008_T1_2D_F4_AnalyzedData.csv	8	1	2	4	
P008_T2_2D_F4_AnalyzedData.csv	8	2	2	4	
P008_T3_2D_F4_AnalyzedData.csv	8	3	2	4	
P008_T4_2D_F4_AnalyzedData.csv	8	4	2	4	
P008_T1_3D_F3_AnalyzedData.csv	8	1	3	3	
P008_T2_3D_F3_AnalyzedData.csv	8	2	3	3	
P008_T3_3D_F3_AnalyzedData.csv	8	3	3	3	
P008_T4_3D_F3_AnalyzedData.csv	8	4	3	3	
P009_T1_2D_F1_AnalyzedData.csv	9	1	2	1	
P009_T2_2D_F1_AnalyzedData.csv	9	2	2	1	
P009_T3_2D_F1_AnalyzedData.csv	9	3	2	1	
P009_T4_2D_F1_AnalyzedData.csv	9	4	2	1	
P009_T1_3D_F2_AnalyzedData.csv	9	1	3	2	
P009_T2_3D_F2_AnalyzedData.csv	9	2	3	2	
P009_T3_3D_F2_AnalyzedData.csv	9	3	3	2	
P009_T4_3D_F2_AnalyzedData.csv	9	4	3	2	
P010_T1_2D_F2_AnalyzedData.csv	10	1	2	2	
P010_T2_2D_F2_AnalyzedData.csv	10	2	2	2	
P010_T3_2D_F2_AnalyzedData.csv	10	3	2	2	
P010_T4_2D_F2_AnalyzedData.csv	10	4	2	2	
P010_T1_3D_F1_AnalyzedData.csv	10	1	3	1	
P010_T2_3D_F1_AnalyzedData.csv	10	2	3	1	
P010_T3_3D_F1_AnalyzedData.csv	10	3	3	1	
P010_T4_3D_F1_AnalyzedData.csv	10	4	3	1	
P011_T1_2D_F4_AnalyzedData.csv	11	1	2	4	
P011_T2_2D_F4_AnalyzedData.csv	11	2	2	4	
P011_T3_2D_F4_AnalyzedData.csv	11	3	2	4	
P011_T4_2D_F4_AnalyzedData.csv	11	4	2	4	
P011_T1_3D_F3_AnalyzedData.csv	11	1	3	3	
P011_T2_3D_F3_AnalyzedData.csv	11	2	3	3	
P011_T3_3D_F3_AnalyzedData.csv	11	3	3	3	
P011_T4_3D_F3_AnalyzedData.csv	11	4	3	3	

### 3.5 Audio_Data.zip

Wav-files are presented per participant (P001-P011), per trial (T1-T4), per condition (2D/3D). Audio files contain 2 tracks (left and right microphone), i.e., they are stereo recordings. Additionally, the data type (Audio), and the performed fragment (F1-F4) are given in the filename. An example of a file name is e.g., ‘P001_T1_2D_F1_Audio.wav’ for a participant (see Table 5).

**Table 5:** Content and file structure of Audio_Data.zip.

File name	Audio_Data.zip				
Content	Participant	Trial	Condition	Piece	Data Type
P001_T1_2D_F2_Audio.csv	1	1	2	2	Audio Data
P001_T2_2D_F2_Audio.csv	1	2	2	2	
P001_T3_2D_F2_Audio.csv	1	3	2	2	
P001_T4_2D_F2_Audio.csv	1	4	2	2	
P001_T1_3D_F1_Audio.csv	1	1	3	1	
P001_T2_3D_F1_Audio.csv	1	2	3	1	
P001_T3_3D_F1_Audio.csv	1	3	3	1	
P001_T4_3D_F1_Audio.csv	1	4	3	1	
P002_T1_2D_F1_Audio.csv	2	1	2	1	
P002_T2_2D_F1_Audio.csv	2	2	2	1	
P002_T3_2D_F1_Audio.csv	2	3	2	1	
P002_T4_2D_F1_Audio.csv	2	4	2	1	
P002_T1_3D_F2_Audio.csv	2	1	3	2	
P002_T2_3D_F2_Audio.csv	2	2	3	2	
P002_T3_3D_F2_Audio.csv	2	3	3	2	
P002_T4_3D_F2_Audio.csv	2	4	3	2	
P003_T1_2D_F3_Audio.csv	3	1	2	3	
P003_T2_2D_F3_Audio.csv	3	2	2	3	
P003_T3_2D_F3_Audio.csv	3	3	2	3	
P003_T4_2D_F3_Audio.csv	3	4	2	3	
P003_T1_3D_F4_Audio.csv	3	1	3	4	
P003_T2_3D_F4_Audio.csv	3	2	3	4	
P003_T3_3D_F4_Audio.csv	3	3	3	4	
P003_T4_3D_F4_Audio.csv	3	4	3	4	
P004_T1_2D_F1_Audio.csv	4	1	2	1	
P004_T2_2D_F1_Audio.csv	4	2	2	1	
P004_T3_2D_F1_Audio.csv	4	3	2	1	
P004_T4_2D_F1_Audio.csv	4	4	2	1	
P004_T1_3D_F2_Audio.csv	4	1	3	2	
P004_T2_3D_F2_Audio.csv	4	2	3	2	
P004_T3_3D_F2_Audio.csv	4	3	3	2	
P004_T4_3D_F2_Audio.csv	4	4	3	2	
P005_T1_2D_F3_Audio.csv	5	1	2	3	
P005_T2_2D_F3_Audio.csv	5	2	2	3	
P005_T3_2D_F3_Audio.csv	5	3	2	3	
P005_T4_2D_F3_Audio.csv	5	4	2	3	
P005_T1_3D_F4_Audio.csv	5	1	3	4	
P005_T2_3D_F4_Audio.csv	5	2	3	4	
P005_T3_3D_F4_Audio.csv	5	3	3	4	
P005_T4_3D_F4_Audio.csv	5	4	3	4	
P006_T1_2D_F1_Audio.csv	6	1	2	1	
P006_T2_2D_F1_Audio.csv	6	2	2	1	
P006_T3_2D_F1_Audio.csv	6	3	2	1	
P006_T4_2D_F1_Audio.csv	6	4	2	1	
P006_T1_3D_F2_Audio.csv	6	1	3	2	
P006_T2_3D_F2_Audio.csv	6	2	3	2	
P006_T3_3D_F2_Audio.csv	6	3	3	2	
P006_T4_3D_F2_Audio.csv	6	4	3	2	
P007_T1_2D_F4_Audio.csv	7	1	2	4	
P007_T2_2D_F4_Audio.csv	7	2	2	4	
P007_T3_2D_F4_Audio.csv	7	3	2	4	
P007_T4_2D_F4_Audio.csv	7	4	2	4	
P007_T1_3D_F3_Audio.csv	7	1	3	3	
P007_T2_3D_F3_Audio.csv	7	2	3	3	
P007_T3_3D_F3_Audio.csv	7	3	3	3	
P007_T4_3D_F3_Audio.csv	7	4	3	3	
P008_T1_2D_F4_Audio.csv	8	1	2	4	
P008_T2_2D_F4_Audio.csv	8	2	2	4	
P008_T3_2D_F4_Audio.csv	8	3	2	4	
P008_T4_2D_F4_Audio.csv	8	4	2	4	
P008_T1_3D_F3_Audio.csv	8	1	3	3	
P008_T2_3D_F3_Audio.csv	8	2	3	3	
P008_T3_3D_F3_Audio.csv	8	3	3	3	
P008_T4_3D_F3_Audio.csv	8	4	3	3	
P009_T1_2D_F1_Audio.csv	9	1	2	1	
P009_T2_2D_F1_Audio.csv	9	2	2	1	
P009_T3_2D_F1_Audio.csv	9	3	2	1	
P009_T4_2D_F1_Audio.csv	9	4	2	1	
P009_T1_3D_F2_Audio.csv	9	1	3	2	
P009_T2_3D_F2_Audio.csv	9	2	3	2	
P009_T3_3D_F2_Audio.csv	9	3	3	2	
P009_T4_3D_F2_Audio.csv	9	4	3	2	
P010_T1_2D_F2_Audio.csv	10	1	2	2	
P010_T2_2D_F2_Audio.csv	10	2	2	2	
P010_T3_2D_F2_Audio.csv	10	3	2	2	
P010_T4_2D_F2_Audio.csv	10	4	2	2	
P010_T1_3D_F1_Audio.csv	10	1	3	1	
P010_T2_3D_F1_Audio.csv	10	2	3	1	
P010_T3_3D_F1_Audio.csv	10	3	3	1	
P010_T4_3D_F1_Audio.csv	10	4	3	1	
P011_T1_2D_F4_Audio.csv	11	1	2	4	
P011_T2_2D_F4_Audio.csv	11	2	2	4	
P011_T3_2D_F4_Audio.csv	11	3	2	4	
P011_T4_2D_F4_Audio.csv	11	4	2	4	
P011_T1_3D_F3_Audio.csv	11	1	3	3	
P011_T2_3D_F3_Audio.csv	11	2	3	3	
P011_T3_3D_F3_Audio.csv	11	3	3	3	
P011_T4_3D_F3_Audio.csv	11	4	3	3	

### 3.6 Questionnaire_Data.zip

The results of 5 standardized questionnaires are presented: the Makransky Multimodal Presence Questionnaire (the social presence subset or MPQS and the physical presence subset or MPQP), the Witmer Presence Questionnaire (WPQ), the Immersive Tendencies Questionnaire (ITQ), the Musical Sophistication Index (MSI), and the Sense of Musical Agency Questionnaire (SOMA). Additionally, demographic data (DQ) were collected, along with some open questions (OQ). The answers to the questionnaires are organized in 3 csv files: ‘MB.csv’, containing answers to the questionnaires presented before the first session (ITQ, MSI and some DQ); and ‘C1.csv’ and ‘C2.csv’, containing the answers to the questionnaires presented before and after each session (MPQS, MPQP, WPQ, SOMA, some DQ and some OQ) in the first and second condition, respectively. A csv file named ‘Legend.csv’ indicates the codes of all the questions, and where the answers to the questions can be found (see Table 6). Since some participants answered in Dutch, all responses were translated to English before adding them to the repository.

**Table 6:** Content and file structure of Questionnaire_Data.zip.

File name	Questionnaire_Data.zip
Content	

C1.csv	questionnaires and answeres of condition 1
C2.csv	questionnaires and answeres of condition 2
MB.csv	questionnaires and answers related to musical background
Legend.csv	questions and question codes

### 3.7 Scores.zip

The scores which were played by both the avatar and the participants are provided, with the correct bowings and articulations. The fragment and the violin section are indicated in the filename. E.g., ‘First_Violin_F2.pdf’, contains the scores of fragment F2, as played by the first violins. See Table 7 for an overview of the file structure and the content.

**Table 7:** Content and file structure of Scores.zip.

File name	Scores.zip
Content	

First_Violin_F1.pdf	Fragment F1 as played by the first violin section
First_Violin_F2.pdf	Fragment F2 as played by the first violin section
Second_Violin_F3.pdf	Fragment F3 as played by the second violin section
Second_Violin_F4.pdf	Fragment F4 as played by the second violin section

### 3.8 Avatar_Data.zip

This directory contains files in csv format with labeled MoCap Data, including data labels. Every column is a data stream from a marker. Marker positions are indicated in Fig. 1. Every marker has 3 data streams, referring to the x, y, and z coordinates of the marker position. In addition to the markers as indicated in Fig. 1, the violin (3–4 markers) and the violin bow (3 markers) are labelled as well. One data file per avatar (First Violin or Second Violin) is presented. Additionally, the data type (MoCap), and the performed fragment (F1-F4) are given in the filename. An example of a file name is e.g., ‘First_Violin_F2_MoCap.csv’ for an avatar (see Table 8).

**Table 8:** Content and file structure of Avatar_Data.zip.

File name	Avatar_Data.zip
Content	
First_Violin_F1_MoCap.csv	MoCap data of the first violin avatar, playing fragment F1
First_Violin_F1_JointAngles.csv	Joint angle data of the first violin avatar, playing fragment F1
First_Violin_F1_Audio.wav	Audio data of the first violin avatar, playing fragment F1
First_Violin_F2_MoCap.csv	MoCap data of the first violin avatar, playing fragment F2
First_Violin_F2_JointAngles.csv	Joint angle data of the first violin avatar, playing fragment F2
First_Violin_F2_Audio.wav	Audio data of the first violin avatar, playing fragment F2
Second_Violin_F3_MoCap.csv	MoCap data of the second violin avatar, playing fragment F3
Second_Violin_F3_JointAngles.csv	Joint angle data of the second violin avatar, playing fragment F3
Second_Violin_F3_Audio.wav	Audio data of the second violin avatar, playing fragment F3
Second_Violin_F4_MoCap.csv	MoCap data of the second violin avatar, playing fragment F4
Second_Violin_F4_JointAngles.csv	Joint angle data of the second violin avatar, playing fragment F4
Second_Violin_F4_Audio.wav	Audio data of the second violin avatar, playing fragment F4

Additionally, the directory contains files in csv format with joint angles, including data labels. Every column is a data stream from a joint (see Fig. 2). Every joint has a varying number of data streams, depending on the calculated angles. In addition to joint angles, the angles of the instrument relative to the body are given as well, the distances of the bow to the bridge, and the distances of the bow to the strings, respectively. One data file per avatar (First Violin or Second Violin) is presented. Additionally, the data type (JointAngles), and the performed fragment (F1-F4) are given in the filename. An example of a file name is e.g., ‘Second_Violin_F3_JointAngles.csv’ for an avatar (see Table 8).

Finally, this directory contains wav-files per avatar (First Violin or Second Violin). Audio files contain 2 tracks (left and right microphone), i.e., they are stereo recordings. Additionally, the data type (Audio), and the performed fragment (F1-F4) are given in the filename. An example of a file name is e.g., ‘First_Violin_F1_Audio.wav’ for an avatar (see Table 8).

## 4 Experimental Design, Materials, and Methods

### 4.1 General protocol

A total of 11 participants were recruited from the Ghent University Student Orchestra (GUSO), a local amateur orchestra. Among them, 6 participants belonged to the first violin section, while 5 participants were part of the second violin section. Each group was assigned to rehearse two different orchestral fragments using an Augmented Reality (AR) environment with a virtual audiovisua representation of a virtual section leader. In this study, there were four fragments in total, labeled as fragments 1, 2, 3, and 4, with two fragments assigned to each violin section.

The participants from the first and second violin sections were randomly divided into two groups: Group 1 and Group 2. Group 1 rehearsed with a 3D avatar for fragments 1 and 3, and a 2D projection of the avatar for fragments 2 and 4. On the other hand, Group 2 rehearsed with a 2D projection of the avatar for fragments 1 and 3, and a 3D avatar for fragments 2 and 4, as indicated in Table 9.

**Table 9:** Overview of participants, their respective violin section and stimulus.

Participant	Violin section	Piece (2D condition)	Piece (3D condition)
1	1	Holst	Dvorak
2	1	Holst	Dvorak
3	2	Dvorak	Brahms
4	1	Dvorak	Holst
5	2	Dvorak	Brahms
6	1	Dvorak	Holst
7	2	Brahms	Dvorak
8	2	Brahms	Dvorak
9	1	Dvorak	Holst
10	1	Holst	Dvorak
11	2	Brahms	Dvorak

Throughout the experiment, the participants took part in four sessions, conducted once per week over the course of one month. The condition (2D or 3D) for each fragment remained consistent within each group. In each trial, the participants followed the same procedure: they practiced the designated fragment with the avatar for 15 min, took a short break, and then performed the fragment while being recorded along with the avatar clip. The participants were instructed to carefully imitate the bowings, dynamics, and articulation of the avatar during the recording.

Motion capture data (MoCap), audio, and video of the participants were recorded during each complete trial, and these datasets were synchronized with the avatar simulations for subsequent analysis. It is worth noting that participants were allowed to freely move around and choose their preferred playing position during practice in both the 2D and 3D conditions, but a fixed position was assigned to them during the recording phase.

Following the recording, participants completed the MPQS, MPQP, WPQ, SOMA, and OQ questionnaires. They then repeated all the aforementioned steps with the other condition and the remaining fragment. Importantly, participants were not allowed to practice the fragments between trials. An outline of the pipeline is given in Campo et al. (see Fig. 2 in [3]). Before the first trial, participants completed the ITQ and MSI questionnaires.

In total, the experiment yielded 11 × 2 × 4 raw datasets, which consisted of MoCap, audio, and questionnaire data, capturing the entire duration of the study.

### 4.2 Participants

Participants had a mean age of 21.3 ± 2.2 years (mean ± SD). All participants in the study had extensive experience playing the violin, with a minimum of 12 years of violin-playing experience (14.7 ± 2.4 years), and had generally spent several years playing in orchestras (3.7 ± 2.3 years). Additionally, the level of musical skill and engagement of the participants was assessed using The Goldsmith Musical Sophistication Index (Gold-MSI), which yielded an average score of 4.9 ± 0.5 [4]. Furthermore, participants' tendency to become immersed in an artificial environment was evaluated by administering a standardized self-reported Immersive Tendencies Questionnaire during the registration process, with an average score of 4.2 ± 0.8 [5] (see Table 10).

**Table 10:** Participant demographics. SD is standard deviation, MSI is the music sophistication index, ITQ is the immersive tendencies questionnaire.

	Age	Age started playing	Years played in orchestra	Years played violin	MSI	ITQ
mean	21.3	6.6	3.7	14.7	5.1	4.2
median	21.0	6.0	3.0	14.0	5.1	4.1
SD	2.2	1.9	2.3	2.4	0.4	0.8
min	18.0	4.0	0.5	12.0	4.3	3.0
max	25.0	10.0	7.5	20.0	5.8	5.7

### 4.3 Questionnaires

We used the standardized self-reported Presence Questionnaire [6] (the Witmer Presence Questionnaire (WPQ)) and two sub-sets of questions from the Multimodal Presence Scale for Virtual Reality [7] (the Makransky Multimodal Presence Questionnaire (MPQS and MPQP) to inquire about participants’ physical and social presence in a Mixed Reality environment. Participants were asked to fill in these questionnaires after every condition in every trial. In total, each participant filled in the WPQ, the MPQS and the MPQP eight times. In addition, after each session, we asked several open-ended questions (OQ) regarding the effectiveness of the training in Mixed Reality setup, regarding the similarity of the experience when practicing with a colleague or with the video at home, and regarding possible application improvements.

Before the experiment, participants filled in the Immersive Tendencies Questionnaire (ITQ), the Musical Sophistication Index (MSI). Additionally, demographic data (DQ) were collected.

### 4.4 HoloLens app

The avatars of the first and second violin were integrated into a HoloLens application, developed using Unity (Unity version 2020.3.2f1, San Francisco, CA, USA). For the 2D condition, the avatar was projected in a frontal view onto a virtual 2D screen (see Fig. 3.c in [3]). In contrast, for the 3D condition, a fully rendered avatar was positioned in front of the participants (see Fig. 3.d in [3]). The performance of the avatar could be controlled by a user interface with start/stop buttons and a slider, allowing for starting, stopping, forwarding, or rewinding of the performance (see Fig. 4 in [3]). The app is not included in the repository.

### 4.5 Data analysis

MoCap data, audio and video data were acquired for every participant. Joint angles of wrist, elbow and shoulder were approximated from the MoCap data using a custom-made MATLAB package [8], based on the standards of the International Society of Biomechanics (ISB) [9], [10], [11], [12]. Additionally, tilting angle and bow position between the bow and the violin was calculated, respectively defined as the distance of the contact point between bow and string to the bridge and frog (see Fig. 2).

### 4.6 Stimulus

The performances of the leaders of the first and second violins from the GUSO were recorded playing two pieces each for the study. The leader of the first violins played Dvorak's Symphony No. 8 in G major, Op. 88, Part III (bars 1–180) and Holst's The Planets, Op. 32: I. Mars (bars 17–83; 95–133; 149–167). The leader of the second violins played Dvorak's Symphony No. 8 in G major, Op. 88, Part I (bars 33–240) and Brahms' Symphony No. 2 in D major, Op. 73, Part III (bars 144–318). These pieces were deliberately chosen to encompass a wide range of techniques and difficulties, with minimal rests. During the recording sessions, the violinists had the option to play along with either a metronome or an orchestral recording through headphones, depending on their preference. The scores of these pieces are also added in the repository.

### 4.7 Motion capture acquisition

Motion capture data were captured using a Qualisys MoCap system with 18 cameras, including 4 RGB cameras (see Fig. 2.a in [3]). MoCap and video data were recorded at a rate of 120 Hz, while audio was recorded using a Y-pair of condenser microphones at 48,000 Hz with a bit depth of 24 bits. All data, including audio, video, and MoCap, were recorded simultaneously and later synchronized using the SMTPE protocol. The motion capture data were then used to generate a whole-body skeleton (see Fig. 2b in [3]), which was used as the basis for modeling and rigging a male avatar (for the first violin) and a female avatar (for the second violin) by the company ARVRtech (ARVRtech, Novi Sad, Serbia).

## CRediT authorship contribution statement

**Adriaan Campo:** Software, Methodology, Conceptualization, Formal analysis, Investigation, Data curation, Writing – original draft, Visualization. **Aleksandra Michałko:** Conceptualization, Investigation, Resources, Project administration. **Bavo Van Kerrebroeck:** Software, Methodology, Conceptualization, Writing – review & editing. **Mark Leman:** Software, Methodology, Conceptualization, Formal analysis, Investigation, Data curation, Writing – original draft, Visualization, Supervision, Funding acquisition.

## Data Availability

Dataset for the assessment of presence and performance in an augmented reality environment for motor imitation learning: a case-study on violinists. (Original data) (zenodo)
